# Lessons from the Field—Two Years of Deploying Operational Wireless Sensor Networks on the Great Barrier Reef

**DOI:** 10.3390/s110706842

**Published:** 2011-06-30

**Authors:** Scott Bainbridge, Damien Eggeling, Geoff Page

**Affiliations:** Australian Institute of Marine Science, PMB 3 MC, Townsville 4810, QLD, Australia; E-Mails: d.eggeling@aims.gov.au (D.E.); g.page@aims.gov.au (G.P.)

**Keywords:** wireless sensor networks, lessons learned, coral reefs, great barrier reef, observing systems, ocean observing, OGC, SWE, WSN

## Abstract

Wireless Sensor Networks promised to do for observation systems what consumer electronics have done for areas like photography—drive down the price per observation (photograph), introduce new functionality and capabilities, and make, what had been a relatively exclusive set of technologies and capabilities, ubiquitous. While this may have been true for some terrestrial sensor networks there are issues in the marine environment that have limited the realization of ubiquitous cheap sensing. This paper reports on the lessons learned from two years of operation of wireless sensor networks deployed at seven coral reefs along the Great Barrier Reef in north-eastern Australia.

## 1. Introduction

In 2007 the Australian Federal and State Governments funded the Australian Integrated Marine Observing System (IMOS) [1] to deploy, over the next four years, a range of ocean observing infrastructure. One component of IMOS was wireless sensor networks to be deployed at seven sites along the Great Barrier Reef (GBR) off north-eastern Australia. The project was seen as a technology test bed for the emerging suite of technologies that comprise wireless sensor networks [2], with the view that the practical lessons learned could be applied to other components of IMOS.

**Figure 1.**
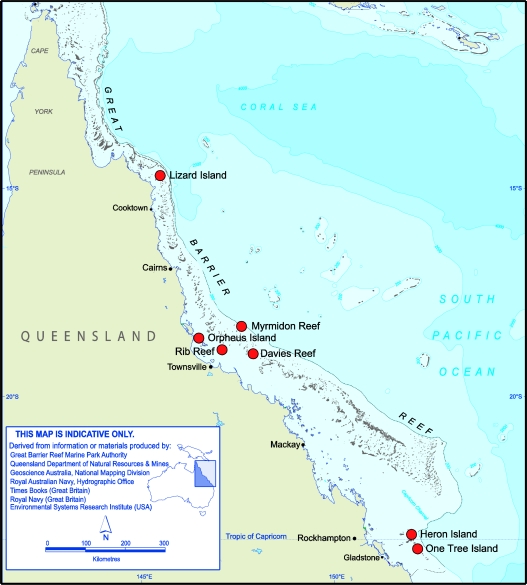
Map of the Sensor Network sites (red dots) along the Great Barrier Reef, Australia.

**Figure 2.**
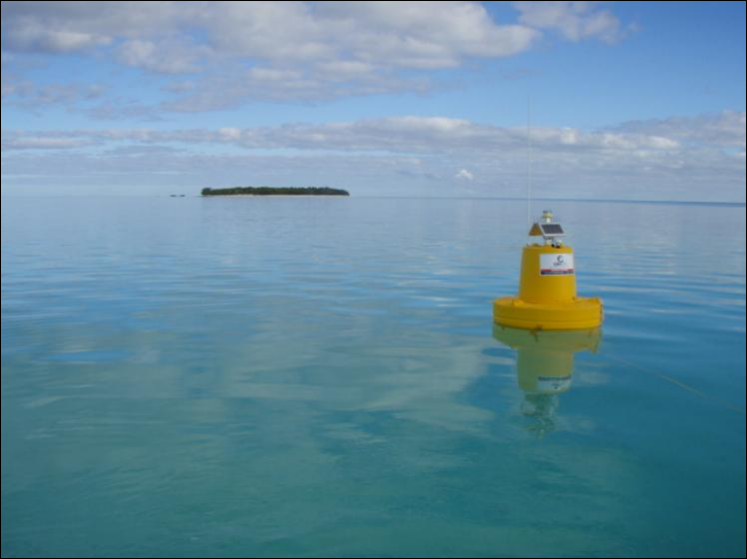
Photograph of a sensor-buoy at Heron Island, southern Great Barrier Reef.

In mid 2008 the first of the seven sites was completed at Heron Island in the southern GBR with the last site deployed in late 2010. The network now consists of seven sites, 33 buoys and towers, 92 instruments delivering some 180 separate data streams with over 19 million observations in just over two years. Figure 1 shows the site locations (red dots) while Figure 2 shows a sensor-buoy at Heron Island. The systems are deployed at reefs up to 100 km offshore and so are isolated and remote.

The project was designed to investigate the use of emerging wireless sensor network technologies to coral reef monitoring with a focus on the factors that contribute to coral bleaching. This includes temperature, light (as PAR—Photosynthetically Active Radiation), salinity and meteorological conditions [3]. The project looked to utilize key technologies, such as real-time two-way Internet Protocol (IP) communication to nodes, the use of cheap sensors deployed in high densities, the use of smart IT systems to allow for adaptive sampling and on-node processing, and the use of cheap consumer grade components, to deploy large scale (hundreds of sensors) cost-effective coral reef observatories.

## 2. The Development of the Great Barrier Reef Ocean Observing System

The reality of actually implementing such a network in the marine environment lead to a number of decisions to re-scope the project, to modify the design and, to a certain degree, the outcomes.

The first change was to deploy traditional oceanographic instruments over untested cheaper sensors. The primary reason for doing this was that the oceanographic community utilizes only a small set of instruments and to deliver scientifically valid observations to this community we needed to use trusted and proven instruments. The second reason was that given the remote nature of the deployments reliability was paramount and so using proven systems reduced the risk of systems failing. The final reason was that the cost of the sensor is only part of the overall cost of the system, again the remote nature of the systems means that a sensor failure can cost tens of thousands of dollars in vessel and staff time so negating any initial saving.

The second change was that there were no commercially available off the shelf “smart” controllers and certainly no implemented standards for such a device. As a result traditional data loggers were used to interface the sensors and to stage the data. The loggers came with data flow and integration software and so this proprietary software was used, along with commercial databases, to monitor, control, task and store the data rather than use open source or standards based systems. The loggers were programmable and so it was possible to implement some of the concepts behind sensor networks, such as adaptive sampling and plug and play, but with considerable effort and in a customized fashion.

As a result we now have a hybrid system using expensive commercial sensors instead of “cheap and cheerful” sensors, using industrial controllers with custom code to partially implement adaptive and other “smart” functionality, such as plug and play, and proprietary software to implement the data flow, data storage and access. This has taken us away from deploying cheap and ubiquitous systems with each data stream (one instrument may produce a number of data streams) costing in the thousands of dollars, not in the hundreds or even tens of dollars initially envisaged. However the system works; delivering some 19 million scientifically valid observations including observations from two cyclones and one tsunami.

## 3. Lessons Learned

Over the life of the project a number of lessons for deploying marine sensor networks have been learned, many the hard way through trial and error. The success of the project has been due to melding the technology components of sensor networks with the experience of the oceanographic community to build a hybrid system using “best of” components. The main lessons learned are detailed below.

### 3.1. Real Time is Hard

A fundamental part of the original vision was real time data. This was one component that survived the rationalization of the original design and has been a fundamental component of what makes the sensor networks different to traditional logged sensors. But real time is difficult for a number of reasons. Firstly any off shore stations are going to have marginal communications, for most of our stations we use 3G phone technologies that we have managed to operate some 80 km out to sea. Issues such as rain and even tide height can interfere with the communications and so, while over long time periods we get reliable communications, over shorter periods there is a high chance the communications will fail. So systems are unreliable over short periods (minutes to hours) but very reliable over longer periods (hours to days).

**Figure 3.**
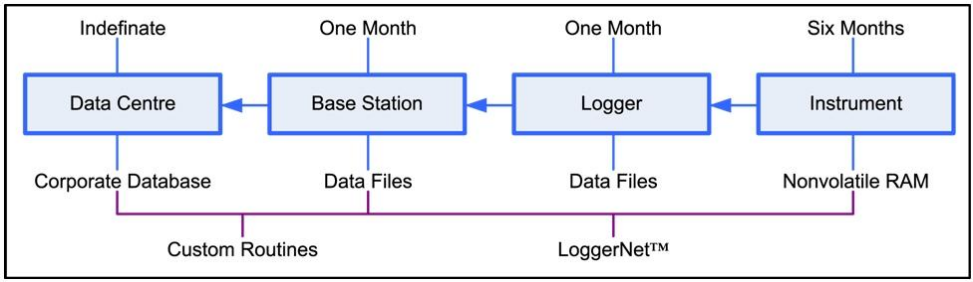
“Store and Forward” data flow schematic showing the components involved, the data storage mechanisms, data storage times (top) and the software used (bottom) to co-ordinate the data flow.

The main solution has been to design each stage of the data flow as a “store and forward” node using the sensor memory and logger hardware to store the data and proprietary software (LoggerNet™ from Campbell Scientific-www.campbellsci.com) to deal with the issue of duplication of data and to co-ordinate the data flow (Figure 3). This latter function is non-trivial and one reason why proprietary commercial loggers and software were used over an open source solution. As a result we have lost very little data with data recovery rates over 98% even though the minute by minute data link uptime can be way below this. The use of a multi-point store and forward architecture along with fault tolerant communications protocols has delivered the required level of data recovery even under adverse conditions. One extreme example was a cyclone that in early 2011 destroyed the surface loggers and communication equipment at one site part-way through the cyclone. The instruments were recovered some weeks later and, as they had stored the data locally, a full record of the event was recovered.

The second part of the solution has been to implement robust communication systems. This includes communication systems that are interrupt tolerant, again using proprietary commercial solutions, along with the store and forward architecture. The other part is the reliability of the communications hardware itself. We had issues with modems that regularly “locked-up” or only had partial links (such as losing their IP settings) that stopped communications. This was solved by implementing a daily power-cycle where the modems were re-set using a switched power supply controlled by the logger.

Other solutions being investigated use a second low-power processor board as a “heartbeat” monitor. This is a small unit, such as a Gumstix™ (www.gumstix.com), that monitors the main data logger and, through serial controlled relays, is able to re-power the main logger and modem. The next step is to have this connected to the modem so that it becomes possible to communicate to the logger via the heartbeat monitor to resolve any issues. Being able to re-set the systems, either automatically via power cycling or through the use of “back-doors”, such as heartbeat monitors, is an important way of dealing with the issue of systems “locking” or becoming unresponsive.

### 3.2. Cheap and Cheerful Doesn’t Work in the Marine Environment

The marine environment represents one of the harshest environments in which to deploy electronic equipment. Apart from the obvious issues of salt water and electronics there are issues of corrosion, especially from electrolysis from dissimilar metals, from the physical action of waves, wind and tides, from exposure to heat and high levels of humidity and the overriding issue of the remoteness of the equipment and the subsequent cost of servicing.

The project design has the equipment serviced every six months with a design goal being double that—that is the equipment is built to last twelve months between servicing. During this time it has to operate with zero critical failures. Unlike terrestrial systems you cannot simply drive to the site and do a quick service and so the effective cost of the failure of a key component is measured in the tens of thousands of dollars.

To deal with this the project has used tried and tested commercial products and in particular it has used mainstream oceanographic components. The moorings that hold the platforms are designed as oceanographic moorings, the connectors are all marine grade connectors which are expensive (up to $50 US per connection), the cables are again marine grade cables and all connections are either made from the same metal or where this is not possible these are insulated and safety-wired into place using plastic zip-ties.

**Figure 4.**
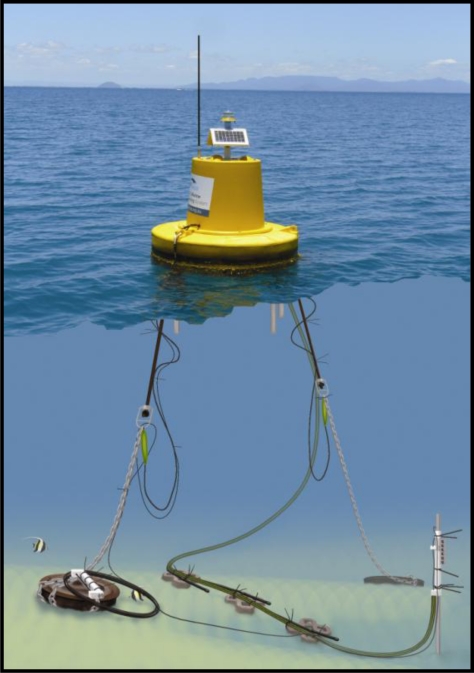
Typical deployment of a sensor-buoy showing the two-point mooring arrangement.

The moorings themselves are built to oceanographic specifications using 250 kg railway wheels as anchors and bungee cords to hold the buoys in place with stainless steel safety wires in case the bungees break. A two point mooring (two anchors placed along the line of typical wave or current direction, see Figure 4) is used to stop the buoy from turning. This, with the bungee cords which are under tension, keeps the buoy stable so that most movement is simple up and down movement with wave action. Having the buoy stable allows for the solar panels to be orientated to best use the available light and stops the sensor cables from becoming entangled. The sensor cables are zip-tied down the stainless steel safety wires which in turn are loosely tied to the bungee cords so they can slide as the bungees move. This design has survived Category-4 Tropical Cyclones and for shallow areas (less than 20 m) has proven to be reliable and effective. A typical mooring is shown in Figure 4.

One of the key issues in designing marine components is that of ‘right-sizing’ the engineering. The larger and more robust components are the better they survive the marine environment but the more they cost and the more difficult they are to deploy, service and recover. The initial idea was that the sensors would be small, cheap and simple but over time there has been a shift to more expensive, heavier equipment. The project has battled against this recognizing that the cost per node is a fundamental constraint on the initial vision of cheap ubiquitous sensing.

For coastal or near-shore deployments the “right-sizing” may be smaller than for remote deployments as the cost to service the equipment is reduced and in some cases the wave energy and potential storm impacts are also reduced. The temptation is to dramatically down-scale the engineering and the quality of the components but our experience is that this needs to be done carefully to ensure that the overall reliability is not compromised. So while near-shore is easier it is still important to design for reliability; investing in reliability during development pays off in increased reliability in the field which ultimately drives down the cost per observation.

### 3.3. Reliability is Everything

Leading on from issue of ‘right-sizing’ the engineering is that of reliability. Reliability is the single hardest outcome to achieve in deploying remote sensors and so the reliability of the end to end system is fundamental to the value it delivers. It is straightforward to built a system that will last a few days but to build one that lasts months, and which produces scientifically valid data, takes more effort.

Two main approaches have been used to ensure reliability. The first is to use proven industry standard components where these exist and the cost is “reasonable”, where the term reasonable is defined as the cheapest set of components that deliver the required service interval.

The second response is to pair technologies or components, especially where new technologies or components are being deployed. One example of this was the use of cheap wireless cards to provide the 802.11 wireless coverage, these were paired with existing proven 900 MHz spread-spectrum communications. The cheap units worked well in the laboratory but failed in the field; as the spread-spectrum radio was still working the data could be routed over this network and the systems re-programmed so no data was lost. Industrial grade 802.11 units, costing ten times as much as the cheaper units, are now being used.

The final component in designing for reliability is to set expectations accordingly. Initially we promised instant access to the real time data from our remote systems but as we moved through a series of equipment failures we down-graded what we could deliver. We now know that systems will fail and so we are better at dealing with, and responding to, outages.

### 3.4. It’s not Easy Being Smart

The initial promise was of “smart” systems that would have some on-node processing capacity, or autonomy, as well as being aware of other nodes in the immediate network [4] and so able to use the network for distributed processing and to implement *ad-hoc* or new models of data flow. The promise included adaptive sampling, that is nodes could change how and what they measure depending on the data they, or other nodes, collect, as well as receiving direct commands from a central data centre.

When the project started there were a number of smart nodes available [5] but no complete “off the shelf” solution that included full data flow and co-ordination software, system monitoring and remote configuration. The need to write considerable software to implement even a basic data flow using these systems along with issues of reliability and power use, meant that the project turned to existing commercial systems selecting a data logger that had been proven over five years of field operations. The loggers came with existing data flow software, data storage functionality along with a full programming language allowing much of the initial base functionality to be quickly implemented. The use of a data logger over a true multi-purpose smart node was a major step away from a true sensor network implementation but reflected the need to quickly deploy reliable proven solutions.

The final component to delivering ‘smart’ systems are the standards to ensure that differing components, levels and systems integrate. While the Open Geospatial Consortium (OGC) Sensor Web Enablement (SWE) [6] standards do include standards for sensor tasking, events and event subscription, these are still under development (V2 of the standards are currently under development) with few robust implementations available, and certainly few that would function at the node level.

The lesson is that there is a significant gap between the types of systems in research laboratories and test deployments and those that are suitable for operational deployment, especially in the marine environment. The lack of standards for many of the ‘smart’ features, or the immaturity of these, limits how this type of functionality can be implemented. The project ended up developing custom software in the loggers to implement some adaptive sampling and ‘plug-and-play’ functionality, but this is a far cry from a standards based implementation.

### 3.5. It has to be Scientifically Valid (It has to Mean Something)

Using the photography analogy, professional photographers would have been appalled at the comparison between their medium format film cameras and the original VGA resolution digital cameras. In the same way the performance of “cheap” sensors in the marine environment is currently a long way behind that of the traditional expensive oceanographic instruments.

The issue is that, unlike a poor quality digital photograph, there is no easy way of knowing if a temperature reading was measured with a fifty cent thermistor or one costing two thousand dollars. If the measurement is to be used in a meaningful way, as a representation of the real world at a certain point in space and time, then the sensor doing the measurement must have an appropriate level of accuracy and precision. Observations need to be made using scientifically valid processes including calibration of sensors before and after deployments, undertaking quality control of the data and understanding the limitations of the sensor, such as how they drift with time, the impact of fouling, and how sampling error changes across the range of measurements.

It is unfortunate that in a rush to push the technology many people forget the basic issues with making any observation. The focus on the technology that makes up the sensor network can over shadow the measurements themselves. In general if the data being collected is to be used independently of the initial study, that is it stands alone as a representation of a real world phenomena, then the system needs to be considered an operational system and so needs to deliver data with the appropriate level of accuracy and precision. The word appropriate here is important, not all studies require three significant digits of precision, but no matter what level is required the accuracy and precision of the sensor needs to be known and documented.

The key lesson is that the data must be fit for purpose and that any deployment must address issues of sensor accuracy and precision and must adopt community appropriate methodologies for sensor calibration, deployment and maintenance.

### 3.6. It has to be Question Driven

Wireless Sensor Networks are often accused of being a technology looking for a question and to a degree this was the case with the Great Barrier Reef deployment. To ensure that the technology delivered scientific outcomes, strong links were developed between the scientific components of the project, especially in developing the sampling design and setting the location of sensors. This was probably not done as well as it should have been at the start of the project but over time the project has worked to re-design the systems based on solid scientific input. By evolving the systems, particular the sampling design and the data products, the network has better aligned its purpose with robust scientific questions and so as it matures is becoming far more question driven than it was initially.

Part of the reason for the slow uptake of the data was the “chicken and egg” problem of engaging users in a system that was new, somewhat untested and which provided a new type of data. Only after two years of data do we have a user-base broad enough to both justify the network and to support future funding and expansion. The need to build a critical mass of data and publications from the data leads to a time delay in gaining a broad user base. This makes the initial part of the project vulnerable as considerable effort is being expended for what looks like little return.

The other issue was that our anticipated user base was not the one that ultimately most strongly engaged with the project. We anticipated that the oceanographic community would use our shallow water reef data as an extension of the deep water data they currently collect. It turns out that most of the issues they wanted to address involved open ocean processes and that the complexity of shallow water reef systems made it hard for them to use our data. The actual user group that did strongly engage were the biologists who were desperate for *in-situ* data to accompany the biological data they were collecting. Issues such as spawning times and behaviors, ranges and locations of fish, interactions between predators and prey, especially sea-birds and the bait-fish they feed on, are all driven to some degree by the small scale local environmental conditions [7]. The sensor network data gives them localized high-resolution data providing an important context for the biological data.

The lesson is there will be a delay between deploying systems and having a queue of users wanting the data just due to the need to collect a critical mass of data before it becomes useful. The other lesson is that there is a need to be actively engaged with the users of the data to ensure that the design, data and data products meets their needs. This has implications for how projects are measured and potentially funded and reviewed.

### 3.7. It has to be End to End

When deploying the Great Barrier Reef Network most of the effort was put into getting the equipment into the water and getting the data coming back. This turned out to be the easy part as the project already had expertise in moorings and marine electronics. The hard part was the data management and access; that is the last mile turned out to be, and still is, the hardest part.

The main issue is that if people can’t find and understand the data then they can’t use it and so we potentially have a project with millions of observations and limited uptake. The challenge is to find ways for people to find and access the data in a manner that makes sense to them. This could be through better data clients, especially for the display and analysis of time series data, through better discovery tools (such as integration into search engines) and the use of new delivery mechanisms such as social networking.

The project has invested heavily in data management as a result. This includes the production of sensor level metadata, in our case to the International Standards Organization (ISO) ISO-19115 [8] standard using the GeoNetwork (http://geonetwork-opensource.org) open source software. It also includes quality control and checking of the data using quality control processes [9] and the documentation of sensor calibration, deployment and maintenance as ancillary documentation.

Much of what we want to do in this area is still outstanding. We would like to have a universal client that allows for the discovery and access of our data (along with other data sets), we would like a better search engine presence so that the biology and general public communities can find our data, and we need to think more about new data products, such as using the social networking systems to advertise and push our data and data products. There is still a large outstanding need to standardize quality control information, to include error information, and to ensure that metadata and other ancillary data (such as calibration data) are linked to the data set to provide a permanent described data resource for future use. Ultimately we need to make our data fit for purpose; this is not easy but essential if the data, and the project, are to have enduring value.

### 3.8. It has to be Maintained

Any system deployed in the marine environment will degrade rapidly due to biological fouling of instruments and the extreme nature of the marine environment. This degradation can be abrupt in the case of storms and cyclones or gradual such as from the effects of waves, tides and wind and the impact of heat, UV light, fouling and so on.

Our experience is that, over a three year period, maintenance costs can be around 50% of the actual original equipment purchase costs. Most equipment has a design life of between three and five years in the marine environment. This means that projects need to budget for substantial on-going maintenance costs of at least half of the initial capital costs over three years and almost 100% over five to six years. This substantially changes funding paradigms as many funding processes are capital focused and so may not cover the substantial on-going maintenance costs.

The other component of maintenance is dealing with unexpected equipment failure. While routine maintenance can deal with the anticipated degradation of equipment there will always be sudden breakages and outages that need to be responded to. If the data streams are to be maintained then spare equipment, service personnel and vessels will all be required at short notice.

The lesson is that the on-going maintenance of any marine system will require resources of at least half that required to do the initial development and deployment. Service intervals need to be in the order of six to twelve months (mostly due to biological fouling) with most equipment only expected to last only three to five years of operation. Finally components will break and there will be outages; responses to these need to be considered and planned before the network is deployed.

### 3.9. It has to Evolve

There is much that we would like to do both to meet our initial design specifications and to take advantage of advances in a range of areas that have occurred over the last few years. The nature of what is planned *versus* what is possible and the tension between these means that systems need to evolve through a series of “generations” to deliver the best possible outcomes for the effort invested. The lesson is to start simple with proven technologies, even if these are known to be outdated when they are deployed, and then to carefully add functionality or components in a controlled and tested manner using industrial solutions where these exist.

To facilitate the evolution of our designs we have established robust test beds using near-shore test equipment that we can cost effectively service and maintain. The other strategy is to pair old and new technologies together so that if one fails we can fall back to the older technology. Any evolution is now done as a staged process with a full set of fall back positions understood and tested.

On the data side the set of OGC and ISO standards are also evolving. We have built our data management system as a series of layers each of which “exposes” itself as a series of web services. By abstracting the interfaces between the layers we can modify the internal programming without disturbing the system as a whole. For example the event detection is currently done by a set of Java routines that query the database, this will probably be replaced by an event detection engine such as Esper (http://esper.codehaus.org). As long as the new system uses and delivers services that conform to the interface standards these changes will not impact the overall functionality of the “stack”.

Sensor Networks are intimately linked into Information Technology and so as opportunities emerge it is essential that they be taken up and utilized, on a reverse case as new observational needs arise we need to find solutions that deliver the required observations. This push-pull tension results in a constant need to evolve what we do and so system evolution is a fundamental component of modern sensor networks and one that needs to be actively managed.

### 3.10. It has to be “Open” (Standards Based)

A follow on from the need to evolve the network is the need to make it as standards based as possible. Adopting an open architecture that uses international standards, where available, increases the ability to adopt new ideas, new standards, and new components into the network. It also allows other groups to use the systems developed and to contribute to a larger effort to develop sensor networks.

The standards that have been adopted include the ISO 19115 spatial metadata standard [8] deployed via the GeoNetwork software. The other standards include the OGC-SWE [6] set of protocols, mostly through the provision of SensorML records for each sensor and through making the data available via Observation & Measurement-ML. To deliver the latter we are using the 52° North Sensor Observation Service (SOS) software (http://52north.org/communities/sensorweb) to deliver OCG-compliant data.

One unexpected outcome from our work has been the high degree of interest from the data development community in the work we are doing. As a result we are involved in a number of informatics projects based on the OGC-SWE [6] protocols, real time data and cloud computing. Being open allows others to participate and facilitates the development of user communities.

## 4. Specific Lessons

The previous section presented some generic lesson learned that can be applied to any marine sensor network deployment. There have also been a number of more specific lessons that have been learned; some relating to specific brands and types of equipment, others to issues around trying to deploy particular types of sensors.

### 4.1. Bio-Fouling with Optical Equipment

Any instrument deployed in the marine environment will become bio-fouled, for many instruments this may interfere with the measurements they make and so render their observations invalid. The obvious example is optical equipment where algae and encrusting organisms may completely cover the instrument within weeks of it being deployed.

**Figure 5.**
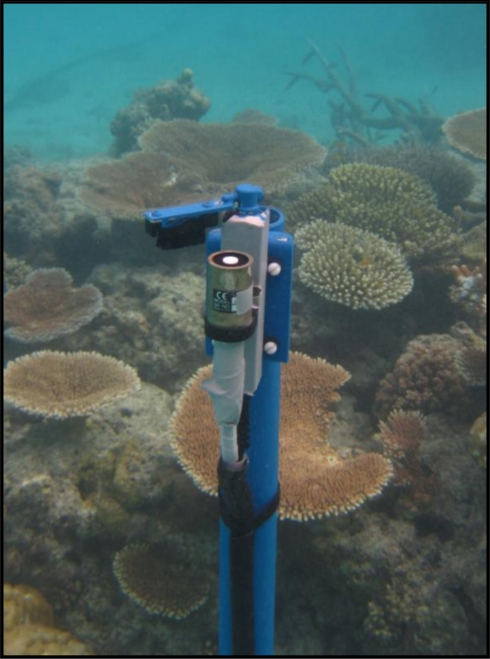
Light Meter installed showing the wiper system in-place.

The project has recently looked to measure above and in-water PAR by deploying a series of light meters (LiCor LI-192: www.licor.com/env/products/light/underwater.jsp). The design includes paired above water and under-water sensors and a second underwater sensor located 1m apart in depth to the first in order to measure water column light attenuation. To deal with bio-fouling a wiper system (Zebra-tech: www.zebra-tech.co.nz/hydro-wipers) was installed to clean the light meters (Figure 5). Experience shows that the wiper is more important as a means to disturb the surface rather than actually clean it, so the wiper does not need to scrape the surface (like a car windscreen wiper) but more brush the surface disturbing anything that is trying to settle.

To measure the effectiveness of the wiper the relationship between the above water sensor (which does not foul in the same way) and the underwater light meters was analyzed. The deployment of two underwater sensors along with a depth sensor means that changes due to water quality and depth can be removed so that any changes in response between the above and in-water sensors can be attributed to bio-fouling. This is not as simple as it sounds, as there can be other factors, but over periods of days and weeks this design will indicate any change in this relationship and hence the degradation in the underwater sensors.

The initial results, over two months, show a 2% decrease in the relationship between the above and below water sensors that can be attributed to bio-fouling; this would likely push out to 6–8% over the six month service interval. A post service calibration will be done to ascertain the real value, in the mean time the real-time data can be corrected for the observed differences between the above and in-water sensors. The lesson is that any optical instrument that is not cleaned on a regular basis needs to have reference sensors so that the impact of bio-fouling or sensor degradation can be measured.

### 4.2. Battery Life with Some Inductive Connected Instruments

The buoys deployed by the project (see Figure 4) work best in shallow protected areas. To get sensors into deeper and less protected areas, such as wave fronts, inductive modem technology from SeaBird Electronics™ (www.seabird.com/products/inductivemodem.htm) was used to communicate between the sensors and the logger. The inductive technology uses specialized modems and couplers to transfer the normal serial signal through a plastic coated mooring cable allowing the instrument to be located up to 7,000 m from the logger, joined only by the mooring cable.

While this technology allows us to position the instruments in deep and rough areas it means that the instruments need to run solely from their inbuilt battery packs and not from surface supplied power. This is not an issue for passive instruments but instruments that incorporate pumps and wipers can draw more power, especially if the pump or wiper is partially obstructed. We found that many of our pumped inductive-modem SeaBird™ SBE37 instruments would only last three to four months on the in-built battery packs, presumably due to excessive power draw from the pump, even though the sampling rate predicted a life of twelve months. The lithium batteries give little indication they are about to fail, so even though we monitored the voltage in real time we were unable to predict failures.

As a result we are converting instruments that are close to the buoys to a standard serial interface that provides surface supplied power. The lesson is that surface supply is preferable to in-built battery packs alone and that instruments with mechanical components can have very different real world power consumption to that detailed by the supplier.

### 4.3. *Ad-Hoc versus* Fixed Networks

One component of true sensor networks [4] is that the network topology is *ad-hoc*; that is the network determines (finds) its own topology. This means that as nodes move the network adjusts to suit. To do this the network needs to determine, normally from message transit times, the topology at any one point. This requires messages to be sent from all nodes to all other nodes and that the message transit times reflect the actual structure of the network.

The issue we had was that, due to wave action and movement of the buoys, there were times when the nodes could not establish the topology due to continued interruption of messages between the buoys. As a result the network would become ‘paralyzed’ and the data transfer would be interrupted.

The solution was to change the buoys to a two point mooring (see Figure 4) to reduce movement, to fix the network topology manually and then to manually update this as required. By manually setting the node topology the network overheads are reduced, the strongest links are automatically used, the effective transmission rate increases with a resulting increase in the overall reliability of the network.

## 5. Conclusions

The initial project was ambitious both in the scope of what it was trying to do and the maturity of the technology that it wanted to use. The success of the project has come from some vicious re-scoping and from a number of lessons learned through two years of full operation. The challenge is to take the initial vision, tempered from the lessons learned, along with the opportunities that have arisen since the project started, to re-focus and renew the project. One key lesson is that ‘cool technology’ should never overshadow the basic process of collecting robust scientifically valid data that has value, is used, and which provides new information and insights.

Technology for technology’s sake is fine for test projects but operational networks need to be grounded in observational science. This is not to ignore the exciting possibilities that technology presents, this still is the fundamental driving force behind the project, but that there needs to be a balance between what actually works in an operational sense *versus* what is the latest and greatest. The real value of the coral reef wireless sensor network project may end up be in facilitating the transition of research grade approaches into operational ones.
